# Crystal structures of μ-oxalato-bis­[azido­(hista­mine)­copper(II)] and μ-oxalato-bis­[(dicyan­amido)(hista­mine)­copper(II)]

**DOI:** 10.1107/S2056989015019908

**Published:** 2015-10-28

**Authors:** Chen Liu, Khalil A. Abboud

**Affiliations:** a Department of Chemistry and Environmental Science, Grenfell Campus, Memorial University of Newfoundland, Corner Brook, Newfoundland, A2H 6P9, Canada; b Department of Chemistry, University of Florida, Gainesville, FL 32611-7200, USA

**Keywords:** crystal structure, dinuclear copper(II) complex, oxalate ligand, histamine ligand, hydrogen bonding

## Abstract

The title compounds, μ-oxalato-κ^4^ *O* ^1^,*O* ^2^:*O* ^1′^,*O* ^2′^-bis­[[4-(2-amino­eth­yl)-1*H*-imid­azole-κ^2^ *N* ^3^,*N* ^4^](azido-κ*N* ^1^)copper(II)], [Cu_2_(C_2_O_4_)(N_3_)_2_(C_5_H_9_N_3_)_2_], (I), and μ-ox­al­ato-κ^4^ *O* ^1^,*O* ^2^:*O* ^1′^,*O* ^2′^-bis­[[4-(2-amino­eth­yl)-1*H*-imidazole-κ^2^ *N* ^3^,*N* ^4^](dicyanamido-κ*N* ^1^)copper(II)], [Cu_2_(C_2_O_4_)(C_2_N_3_)_2_(C_5_H_9_N_3_)_2_], (II), are two oxalate-bridged dinuclear copper complexes. Each Cu^II^ ion adopts a five-coordinate square-pyramidal coordination sphere where the basal N_2_O_2_ plane is formed by two O atoms of the oxalate ligand and two N atoms of a bidentate chelating histamine mol­ecule. The apical coordination site in compound (I) is occupied by a monodentate azide anion through one of its terminal N atoms. The apical coordination site in compound (II) is occupied by a monodentate dicyanamide anion through one of its terminal N atoms. The mol­ecules in both structures are centrosymmetric. In the crystals of compounds (I) and (II), the dinuclear complexes are linked through N—H⋯*X* and C—H⋯*X* (*X* = N, O) hydrogen bonds where the donors are provided by the histamine ligand and the acceptor atoms are provided by the azide, dicyanamide, and oxalate ligands. In compound (I), the coordinatively unsaturated copper ions inter­act with the histamine ligand *via* a C—H⋯Cu inter­action. The coordinatively unsaturated copper ions in compound (II) inter­act *via* a weak N⋯Cu inter­action with the dicyanamide ligand of a neighboring mol­ecule. The side chain of the histamine ligand is disordered over three sets of sites in (II).

## Abstract 2

The title compounds are two closely related oxalate-bridged dinuclear copper complexes. The histamine ligand is in a gauche conformation and coordinates to copper ions in a bidentate chelating fashion. The dinuclear complexes are linked to form three-dimensional networks *via* different types of hydrogen-bond and weak inter­actions.

## Chemical context   

The oxalate ligand often plays an important role as a versatile bridging ligand in the formation of coordination polymers of various dimensionalities, including dinuclear complexes, chains, two-dimensional layered structures *etc*. (Coronado *et al.*, 2003 ▸; Pardo *et al.*, 2010 ▸). The oxalate dianion can coordinate to two metal ions in a bis-bidentate fashion to form a dinuclear unit, although other coordination modes of oxalate have also been reported (Hernández-Molina *et al.*, 2001 ▸). In our effort to design and synthesize coordination polymers in a more rational and controlled fashion, we decided to use oxalate-based dinuclear complexes as mol­ecular building blocks in preparing ladder-like coordination polymers. One strategy would be to introduce a linear bridging ligand to link the dinuclear units into ladder-like structures. Some potential choices of linear bridging ligands include azide and dicyanamide anions which have been widely used as bridging ligands in the design and synthesis of coordination polymers. The azide anion mainly coordinates in an end-on or end-to-end fashion (Escuer & Aromí, 2006 ▸; Stamatatos & Christou, 2009 ▸), while dicyanamide exhibits several different coordination modes (Batten & Murray, 2003 ▸). During our attempts to react azide and dicyanamide with oxalate-bridged bis­copper(II) complexes, we obtained the title compounds as dinuclear units inter­acting *via* hydrogen-bonding and weak inter­actions.
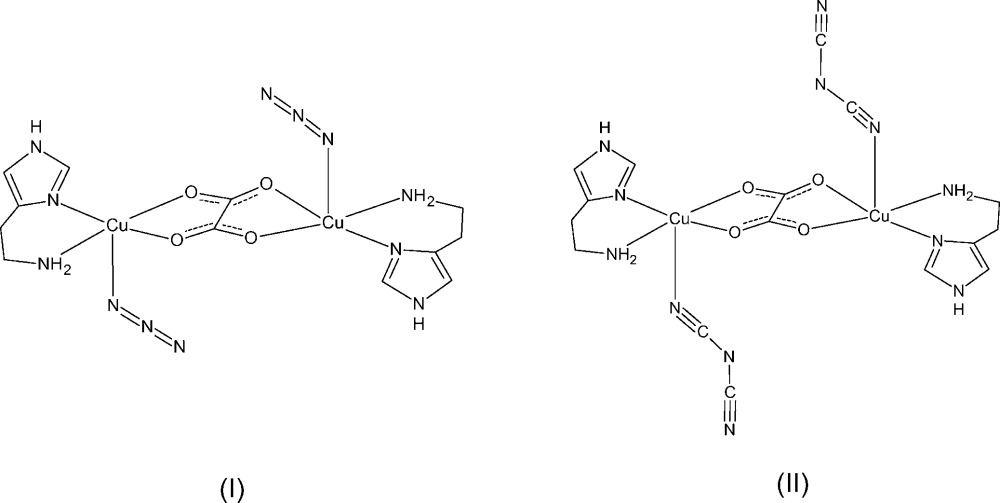


## Structural commentary   

**Figure 1:**
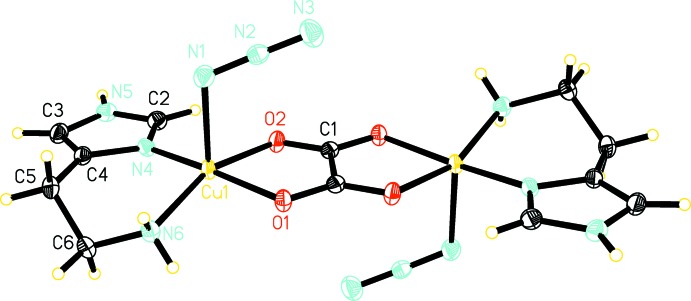
The mol­ecular structure of compound (I). Displacement ellipsoids are drawn at the 50% probability level. Unlabeled atoms are related by inversion symmetry (−*x* + 1, −*y* + 1, −*z* + 1).

**Figure 2:**
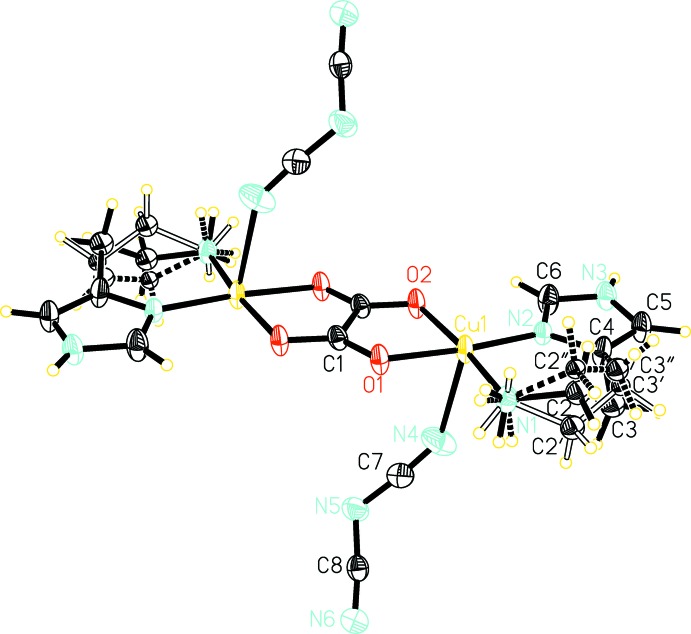
The mol­ecular structure of compound (II). Displacement ellipsoids are drawn at the 50% probability level. Unlabeled atoms are related by inversion symmetry (−*x* + 1, −*y* + 1, −*z*). All disordered components are shown.

Compound (I) crystallizes in the ortho­rhom­bic space group *Pbca* (Fig. 1 ▸) and compound (II) crystallizes in the monoclinic space group *P*2_1_/*c* (Fig. 2 ▸). Both complexes are binuclear with a bridging oxalate anion coordinating in a bis-bidentate fashion to two copper cations, and both binuclear complexes are centrosymmetric with a center of inversion located at the center of the bridging oxalate anion. The copper ions in both compounds have a five-coordinate square-pyramidal coordination geometry. In compound (I), the basal N_2_O_2_ plane defined by N4, N6, O1, and O2 has an r.m.s. deviation of 0.116 Å and the Cu1 atom is displaced from this basal plane toward the apical site by 0.240707 (11) Å. In compound (II), the basal N_2_O_2_ plane defined by N1, N2, O1, and O2 has an r.m.s. deviation of 0.023 Å and the Cu1 atom is displaced from this basal plane by 0.0274291 (17) Å. The four Cu—O and Cu—N bonds in the basal plane have similar lengths in both of the title compounds, with the Cu—O bonds being slightly longer than the Cu—N bonds. These bond length values are in good agreement with those reported for other oxalate-bridged dinuclear copper complexes (Felthouse *et al.*, 1976 ▸; Gleizes *et al.*, 1992 ▸; Mukherjee *et al.*, 2004 ▸; Zhang *et al.*, 2012 ▸). The apical coordination site of the copper ions is occupied by a monodentate nonbridging azide anion in compound (I) and by a monodentate nearly bridging (see below) dicyanamide anion in compound (II). The apical Cu—N bond in both title compounds is significantly longer than the Cu—O and Cu—N bonds in the basal plane.

The distance between the two Cu^II^ ions bridged by oxalate is 5.24755 (18) Å in compound (I) and 5.2151 (3) Å in compound (II). These distances are within the typical range of values for oxalate-bridged dinuclear copper complexes (Felthouse *et al.*, 1976 ▸; Gleizes *et al.*, 1992 ▸; Mukherjee *et al.*, 2004 ▸; Xu, 2011 ▸).

In both of the title compounds, the histamine mol­ecule adopts the N^τ^—H tautomer form where imidazole atom N5 in compound (I) and N3 in compound (II) are protonated. The histamine ligand coordinates to the copper ion in a bidentate chelating fashion *via* the nonprotonated N atom on the imidazole ring as well as the N atom on the ethyl­amino side chain, resulting in a *gauche* conformation for the histamine ligand.

## Supra­molecular features   

In the crystals of the title compounds, hydrogen-bonding and weak inter­actions exist between the dinuclear complexs. As a result, the dinuclear complexes are linked to form rows which then assemble into sheets, and finally sheets stack to form three-dimensional networks.

**Figure 3:**
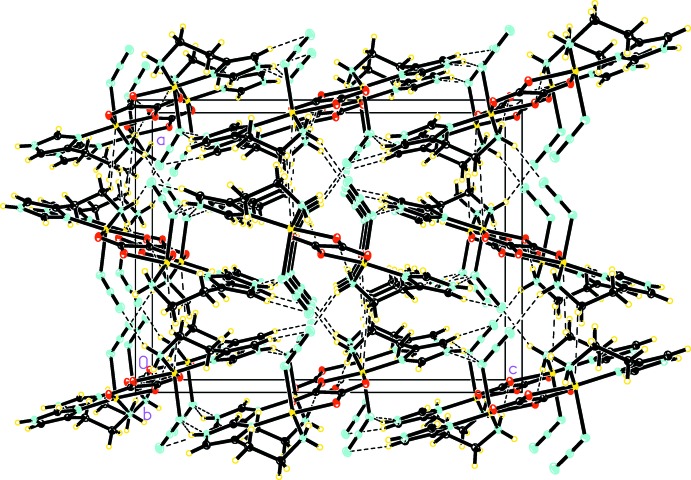
The crystal packing of compound (I), showing the hydrogen bonds as dashed lines.

**Figure 4:**
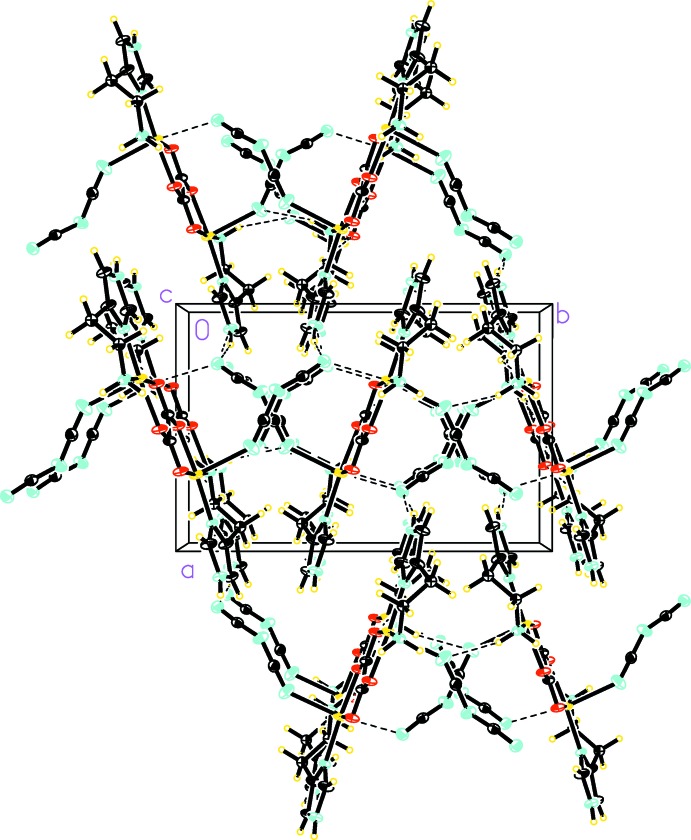
The crystal packing of compound (II), showing the hydrogen bonds as dashed lines. Atoms of disordered components have been omitted for clarity.

The two title compounds exhibit a common set of hydrogen bonds between dinuclear complexes where the histamine mol­ecule is the sole source of hydrogen-bond donors. The NH_2_ group of the histamine ethyl­amino side chain functions as a donor to form an N—H⋯O hydrogen bond where the acceptor is the O1 atom of the oxalate ligand. This hydrogen bond results in the formation of rows of parallel dinuclear complexes along the crystallographic *b* axis in compound (I) and along the crystallographic *c* axis in compound (II). Within each row, dinuclear complexes are placed side-to-side with each other rather than stacking directly above and below each other. As a result, there is essentially no overlap between the dinuclear planes. The same NH_2_ group of histamine is also the donor for a N—H⋯N hydrogen bond where the acceptor is the N3 atom of the azide in compound (I) and N4 of the dicyanamide in compound (II). This hydrogen bond links rows of dinuclear complexes to form sheets parallel to the *ab* plane in compound (I) (Fig. 3 ▸) and to the *bc* plane in compound (II) (Fig. 4 ▸). The protonated N—H group on the imidazole ring is another hydrogen-bond donor for a second N—H⋯N hydrogen bond, where the acceptor is N1 of azide in compound (I) and N6 of dicyanamide in compound (II). This hydrogen bond operates between dinuclear complexes from neighboring sheets and assembles the sheets into a three-dimensional network. For numerical values and symmetry operators for (I) and (II), see Tables 2 ▸ and 3 ▸.

In addition to the traditional N—H⋯*X* (*X* = N, O) hydrogen bonds, both title compounds also exhibit a weak C—H⋯*X* (*X* = N, O) hydrogen bond between neighboring dinuclear complexes. In compound (I), this weak hydrogen bond is C3—H3⋯N3^v^ where the donor is the C—H group on the imidazole ring and the acceptor is N3 of the azide. The C3—H3⋯N3^v^ hydrogen bonds (Table 2 ▸) operate between dinuclear complexes from different sheets and link sheets to form a three-dimensional network. In compound (II), this weak hydrogen bond is C2—H2*A*⋯O2^iv^ (Table 3 ▸), where the donor is an aliphatic C—H group on the ethyl­amino side chain of histamine and the acceptor is the O2^iv^ of oxalate. The C2—H2*A*⋯O2^iv^ bonds operate between dinuclear complexes in the same row. Weak hydrogen bonds of the C—H⋯*X* (*X* = N, O) types are prevalent in crystal structures and are formed with many different types of acceptor. The geometrical features of these weak hydrogen bonds exhibit a wide range of variation depending on the strength of the donors and acceptors. The values of the bond lengths and angles for the two title compounds in this study are within the typical range for C—H⋯*X* (*X* = N, O) hydrogen bonds (Mascal, 1998 ▸; Sigel *et al.*, 1998 ▸; Janiak & Scharmann, 2003 ▸; Youm *et al.*, 2006 ▸).

In compound (I), the coordinatively unsaturated copper ions inter­act with the histamine ligand *via* C6—H6*C*/H6*D*⋯Cu1^vi^ [symmetry code: (vi) 1/2-*x*, −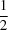 + *y*, *z*] inter­actions (Braga *et al.*, 1998 ▸). These inter­actions exist along the *a* axis between neighboring rows of dinuclear complexes. The H6*C*/H6*D*⋯Cu1^vi^ distances are 3.14625 (16) and 3.19821 (12) Å for H6*C* and H6*D*, respectively. The C6⋯Cu1^vi^ separation is 3.64696 (16) Å. These distances are significantly longer than those found in the traditional and weak hydrogen bonds described above. The C6—H6*C*/H6*D*⋯Cu1^vi^ angles are 112.8720 (3) and 109.287 (4)° for H6*C* and H6*D*, respectively. These distances and bond angle values are in good agreement with other similar inter­actions found in the literature (Brookhart & Green, 1983 ▸; Braga *et al.*, 1998 ▸; Yang *et al.*, 2004 ▸; Yamauchi *et al.*, 2008 ▸).

In compound (II), the dicyanamide ligands are largely perpendicular to the dinuclear plane, making it possible for the coordinatively unsaturated copper ions to inter­act directly with the terminal noncoordinating N6 atom of the dicyanamide ligand of a neighboring dinuclear complex. The N6⋯Cu1^v^ [symmetry code: (v) 1-*x*, 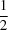 + *y*, 1/2-*z*] distance is 2.60123 (18) Å, indicating a much stronger inter­action than the C—H⋯Cu inter­action in compound (I). Similar to the C—H⋯Cu inter­actions in compound (I), the N6⋯Cu1^v^ inter­actions in compound (II) also operate between neighboring rows of dinuclear complexes along the *b* axis.

## Synthesis and crystallization   

Compound (I) was synthesized by mixing copper(II) perchlorate hexa­hydrate (1.0 mmol), histamine di­hydro­chloride (1.0 mmol), sodium oxalate (0.5 mmol), and sodium azide (1.0 mmol) in deionized water (25 ml) to form an aqueous solution. The solution was allowed to stand in air. After a few days, dark-green prismatic crystals were collected, washed with deionized water, and dried in air (yield 63%). Selected IR (KBr, cm^−1^): 3271, 3228 (primary amine N—H), 2041 (N=N), 1637 (C—O), 1585 (C=C), 1078 (imidazole C—N). Elemental analysis calculated for C_12_H_18_Cu_2_N_12_O_4_: C 27.64, H 3.48, N 32.24%. Found: C 27.53, H 3.17, N 32.42%.

Compound (II) was synthesized in a similar manner, except that the sodium azide was replaced by sodium dicyanamide (1.0 mmol). After a few days, deep-blue plates of crystals were collected, washed with deionized water, and dried in air (yield 55%). Selected IR (KBr, cm^−1^): 3296, 3253 (primary amine N—H), 2254, 2204, 2146 (C≡N), 1646 (C—O), 1571 (C=C), 1079 (imidazole C—N). Elemental analysis calculated for C_16_H_18_Cu_2_N_12_O_4_: C 33.74, H 3.18, N 29.51%. Found: C 33.59, H 2.90, N 29.79%.

## Refinement details   

Crystal data, data collection and structure refinement details are summarized in Table 1 ▸. All H atoms, except the amine protons, were placed in geometrically idealized positions and allowed to ride on their parent atoms, with C—H = 0.93/1.00 Å and *U*_iso_(H) = 1.2/1.5*U*_eq_(C). Methyl H atoms were allowed to rotate around the corresponding C—C bond. In compound (II), the C_2_H_4_ unit of the histamine side chain is disordered and was refined anisotropically over three positions with their site-occupation factors constrained to unity. Equivalent bond lengths were restrained to be similar [SAME command in *SHELXL2014* (Sheldrick, 2015 ▸), s.u. = 0.005 Å], and disordered atoms were subjected to a rigid bond restraint (RIGU command in *SHELXL2014*, s.u. = 0.004 Å^2^). As a consequence of the disorder, the two protons on the adjacent N atom are disordered and also were included in three idealized positions and were treated riding on their parent atoms. The other amino protons were obtained from difference Fourier maps and refined freely. In compound (I), the largest residual difference desnity peak (0.78 e Å^−3^) found at (0.3411, 0.4069, 0.9096) could be due to a minor alternative position for an additional Cu^II^ ion. Due to its small size, copper disorder was not included in the final refinement model.

## Supplementary Material

Crystal structure: contains datablock(s) I, II, global. DOI: 10.1107/S2056989015019908/zl2645sup1.cif

Structure factors: contains datablock(s) I. DOI: 10.1107/S2056989015019908/zl2645Isup5.hkl

Structure factors: contains datablock(s) II. DOI: 10.1107/S2056989015019908/zl2645IIsup6.hkl

CCDC references: 1432483, 1432482

Additional supporting information:  crystallographic information; 3D view; checkCIF report

## Figures and Tables

**Table 1:** Hydrogen-bond geometry (Å, °) for (I)

*D*—H⋯*A*	*D*—H	H⋯*A*	*D*⋯*A*	*D*—H⋯*A*
N5—H5⋯N1^i^	0.88	2.18	2.904 (2)	140
N5—H5⋯N3^ii^	0.88	2.69	3.258 (2)	124
N6—H6*A*⋯N3^iii^	0.87 (3)	2.29 (3)	3.099 (3)	154 (2)
N6—H6*B*⋯O1^iv^	0.89 (3)	2.17 (3)	3.061 (2)	175 (2)
C3—H3⋯N3^v^	0.95	2.54	3.476 (8)	169

Symmetry codes: (i) 
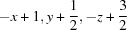
; (ii) 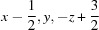; (iii) 
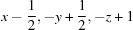
; (iv) 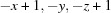; (v) 
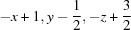
.

**Table 2:** Hydrogen-bond geometry (Å, °) for (II)

*D*—H⋯*A*	*D*—H	H⋯*A*	*D*⋯*A*	*D*—H⋯*A*
N1—H1*E*⋯O1^i^	0.91	2.29	3.131 (3)	154
N1—H1*F*⋯N4^ii^	0.91	2.50	3.377 (4)	161
N3—H3⋯N6^iii^	0.91 (5)	2.10 (5)	2.954 (3)	157 (4)
C2—H2*A*⋯O2^iv^	0.99	2.52	3.277 (1)	133

Symmetry codes: (i) 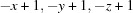; (ii) 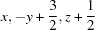; (iii) 
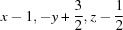
; (iv) 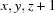.

**Table 3:** Experimental details

	(I)	(II)
Crystal data		
Chemical formula	[Cu_2_(C_2_O_4_)(N_3_)_2_(C_5_H_9_N_3_)_2_]	[Cu_2_(C_2_O_4_)(C_2_N_3_)_2_(C_5_H_9_N_3_)_2_]
*M*_r_	521.46	569.50
Crystal system, space group	Orthorhombic, *P* *b* *c* *a*	Monoclinic, *P*2_1_/*c*
Temperature (K)	173	173
*a*, *b*, *c* (Å)	13.4419 (7), 7.4576 (4), 17.7662 (9)	9.6816 (7), 14.7236 (11), 7.4604 (6)
α, β, γ (°)	90, 90, 90	90, 90.112 (1), 90
*V* (Å^3^)	1780.96 (16)	1063.46 (14)
*Z*	4	2
Radiation type	Mo *K*α	Mo *K*α
μ (mm^−1^)	2.44	2.05
Crystal size (mm)	0.17 × 0.11 × 0.11	0.32 × 0.27 × 0.18

Data collection		
Diffractometer	Bruker SMART CCD area detector	Bruker SMART CCD area detector
Absorption correction	Integration (*SADABS*; Bruker, 1998 ▸)	Analytical (*SADABS*; Bruker, 1998 ▸)
*T*_min_, *T*_max_	0.682, 0.835	0.340, 0.503
No. of measured, independent and observed [*I* > 2σ(*I*)] reflections	10138, 2027, 1768	6331, 2374, 2172
*R*_int_	0.061	0.061
(sin θ/λ)_max_ (Å^−1^)	0.650	0.650

Refinement		
*R*[*F* ^2^ > 2σ(*F* ^2^)], *wR*(*F* ^2^), *S*	0.026, 0.070, 1.08	0.034, 0.096, 1.15
No. of reflections	2027	2374
No. of parameters	145	198
No. of restraints	0	55
H-atom treatment	H atoms treated by a mixture of independent and constrained refinement	H atoms treated by a mixture of independent and constrained refinement
Δρ_max_, Δρ_min_ (e Å^−3^)	0.78, −0.26	0.34, −0.42

Computer programs: *SMART* and *SAINT* (Bruker, 1998 ▸), *SHELXS97* and *SHELXTL-Plus* (Sheldrick, 2008 ▸), *SHELXL2014* (Sheldrick, 2015 ▸) and *publCIF* (Westrip, 2010 ▸).

## supplementary crystallographic information

### (I) µ-Oxalato-κ4O1,O2:O1',O2'-bis[[4-(2-aminoethyl)-1H-imidazole-κ2N3,N4](azido-κN1)copper(II)] Crystal data

**Table:**

[Cu_2_(C_2_O_4_)(N_3_)_2_(C_5_H_9_N_3_)_2_]	*D*_x_ = 1.945 Mg m^−^^3^
*M**_r_* = 521.46	Mo *K*α radiation, λ = 0.71073 Å
Orthorhombic, *P**b**c**a*	Cell parameters from 88 reflections
*a* = 13.4419 (7) Å	θ = 2.0–28.0°
*b* = 7.4576 (4) Å	µ = 2.44 mm^−^^1^
*c* = 17.7662 (9) Å	*T* = 173 K
*V* = 1780.96 (16) Å^3^	Prism, green
*Z* = 4	0.17 × 0.11 × 0.11 mm
*F*(000) = 1056	

### (I) µ-Oxalato-κ4O1,O2:O1',O2'-bis[[4-(2-aminoethyl)-1H-imidazole-κ2N3,N4](azido-κN1)copper(II)] Data collection

**Table:**

Bruker SMART CCD area-detector diffractometer	2027 independent reflections
Radiation source: fine-focus sealed tube	1768 reflections with *I* > 2σ(*I*)
Graphite monochromator	*R*_int_ = 0.061
ω scans	θ_max_ = 27.5°, θ_min_ = 2.3°
Absorption correction: integration (SADABS; Bruker, 1998)	*h* = −13→17
*T*_min_ = 0.682, *T*_max_ = 0.835	*k* = −9→9
10138 measured reflections	*l* = −20→23

### (I) µ-Oxalato-κ4O1,O2:O1',O2'-bis[[4-(2-aminoethyl)-1H-imidazole-κ2N3,N4](azido-κN1)copper(II)] Refinement

**Table:**

Refinement on *F*^2^	Secondary atom site location: structure-invariant direct methods
Least-squares matrix: full	Hydrogen site location: mixed
*R*[*F*^2^ > 2σ(*F*^2^)] = 0.026	H atoms treated by a mixture of independent and constrained refinement
*wR*(*F*^2^) = 0.070	*w* = 1/[σ^2^(*F*_o_^2^) + (0.0279*P*)^2^ + 1.2918*P*] where *P* = (*F*_o_^2^ + 2*F*_c_^2^)/3
*S* = 1.08	(Δ/σ)_max_ = 0.002
2027 reflections	Δρ_max_ = 0.78 e Å^−^^3^
145 parameters	Δρ_min_ = −0.26 e Å^−^^3^
0 restraints	Extinction correction: SHELXL2014 (Sheldrick, 2015), Fc^*^=kFc[1+0.001xFc^2^λ^3^/sin(2θ)]^-1/4^
Primary atom site location: structure-invariant direct methods	Extinction coefficient: 0.0022 (3)

### (I) µ-Oxalato-κ4O1,O2:O1',O2'-bis[[4-(2-aminoethyl)-1H-imidazole-κ2N3,N4](azido-κN1)copper(II)] Special details

**Table:**

Geometry. All e.s.d.'s (except the e.s.d. in the dihedral angle between two l.s. planes) are estimated using the full covariance matrix. The cell e.s.d.'s are taken into account individually in the estimation of e.s.d.'s in distances, angles and torsion angles; correlations between e.s.d.'s in cell parameters are only used when they are defined by crystal symmetry. An approximate (isotropic) treatment of cell e.s.d.'s is used for estimating e.s.d.'s involving l.s. planes.
Refinement. Refinement of *F*^2^ against ALL reflections. The weighted *R*-factor *wR* and goodness of fit *S* are based on *F*^2^, conventional *R*-factors *R* are based on *F*, with *F* set to zero for negative *F*^2^. The threshold expression of *F*^2^ > σ(*F*^2^) is used only for calculating *R*-factors(gt) *etc*. and is not relevant to the choice of reflections for refinement. *R*-factors based on *F*^2^ are statistically about twice as large as those based on *F*, and *R*- factors based on ALL data will be even larger.All H atoms were positioned geometrically (C—H = 0.93/1.00 Å) and allowed to ride with *U*_iso_(H)= 1.2/1.5*U*_eq_(C). Methyl ones were allowed to rotate around the corresponding C—C.The amino protons were obtained from a Difference Fourier map and refined freely.A small peak (0.78) was found at 0.3411 0.4069 0.9096, which had the following geometry around it: We believe it is a small trace of a Cu center but was not included in the final refinement model for its small size.ENVIRONMENT OF Q1Ligand Symcode Dist. Angles symm operationO1 6566 2.581 *x*. 5 - *y*. 5 + *z* N1 5656 2.039 107.5 1 - *x*. 5 + *y* 1.5 - *z* N2 5656 2.580 119.2 26.6 1 - *x*. 5 + *y* 1.5 - *z* N3 4557 1.902 108.9 127.6 101.4 -.5 + *x y*. 5 - *z* N5 1555 1.928 97.9 94.0 113.7 116.5

### (I) µ-Oxalato-κ4O1,O2:O1',O2'-bis[[4-(2-aminoethyl)-1H-imidazole-κ2N3,N4](azido-κN1)copper(II)] Fractional atomic coordinates and isotropic or equivalent isotropic displacement parameters (Å^2^)

**Table:**

	*x*	*y*	*z*	*U*_iso_*/*U*_eq_	
Cu1	0.44296 (2)	0.24198 (3)	0.59064 (2)	0.01472 (11)	
O1	0.47496 (11)	0.27517 (17)	0.48108 (7)	0.0173 (3)	
O2	0.46843 (11)	0.50801 (17)	0.59516 (7)	0.0181 (3)	
N1	0.60261 (13)	0.1548 (2)	0.60977 (10)	0.0221 (4)	
N2	0.65931 (13)	0.2515 (2)	0.57879 (9)	0.0182 (3)	
N3	0.71372 (14)	0.3492 (3)	0.54767 (10)	0.0301 (4)	
N4	0.39863 (12)	0.23846 (19)	0.69487 (9)	0.0159 (3)	
N5	0.36694 (12)	0.3189 (2)	0.81078 (9)	0.0199 (3)	
H5	0.3614	0.3858	0.8514	0.024*	
N6	0.37467 (14)	0.0143 (2)	0.56327 (9)	0.0182 (3)	
H6A	0.3427 (19)	0.035 (3)	0.5215 (14)	0.025 (6)*	
H6B	0.420 (2)	−0.065 (4)	0.5489 (14)	0.027 (6)*	
C1	0.49805 (13)	0.5671 (2)	0.53301 (10)	0.0156 (4)	
C2	0.39970 (14)	0.3740 (3)	0.74353 (10)	0.0187 (4)	
H2A	0.4207	0.4926	0.7322	0.022*	
C3	0.34345 (15)	0.1401 (3)	0.80562 (11)	0.0203 (4)	
H3A	0.3182	0.0661	0.8447	0.024*	
C4	0.36342 (14)	0.0895 (2)	0.73347 (10)	0.0164 (4)	
C5	0.35239 (15)	−0.0881 (2)	0.69514 (11)	0.0187 (4)	
H5A	0.3107	−0.1673	0.7268	0.022*	
H5B	0.4188	−0.1445	0.6903	0.022*	
C6	0.30567 (14)	−0.0729 (3)	0.61751 (11)	0.0188 (4)	
H6C	0.2882	−0.1941	0.5990	0.023*	
H6D	0.2436	−0.0019	0.6210	0.023*	

### (I) µ-Oxalato-κ4O1,O2:O1',O2'-bis[[4-(2-aminoethyl)-1H-imidazole-κ2N3,N4](azido-κN1)copper(II)] Atomic displacement parameters (Å^2^)

**Table:**

	*U*^11^	*U*^22^	*U*^33^	*U*^12^	*U*^13^	*U*^23^
Cu1	0.01840 (16)	0.01299 (14)	0.01277 (15)	−0.00233 (8)	0.00262 (8)	0.00036 (8)
O1	0.0229 (7)	0.0142 (6)	0.0148 (6)	−0.0025 (5)	0.0022 (5)	0.0010 (5)
O2	0.0246 (7)	0.0158 (6)	0.0140 (6)	−0.0019 (5)	0.0021 (5)	0.0016 (5)
N1	0.0205 (9)	0.0218 (8)	0.0240 (8)	0.0025 (7)	0.0006 (7)	0.0050 (7)
N2	0.0192 (8)	0.0197 (8)	0.0156 (7)	0.0012 (6)	−0.0015 (7)	−0.0017 (6)
N3	0.032 (1)	0.0341 (10)	0.0243 (9)	−0.0096 (8)	0.0050 (8)	−0.0009 (8)
N4	0.0156 (8)	0.0166 (8)	0.0155 (8)	−0.0024 (6)	0.0020 (6)	0.0001 (6)
N5	0.0212 (8)	0.0242 (8)	0.0143 (8)	0.0004 (7)	0.0006 (7)	−0.0039 (6)
N6	0.0220 (8)	0.0173 (8)	0.0152 (8)	−0.0018 (7)	0.0017 (7)	−0.0013 (6)
C1	0.0149 (8)	0.0159 (9)	0.0158 (9)	0.0004 (7)	−0.0012 (7)	0.0006 (7)
C2	0.0198 (9)	0.0190 (9)	0.0172 (9)	−0.0007 (7)	−0.0003 (7)	−0.0016 (7)
C3	0.0199 (9)	0.0235 (10)	0.0174 (9)	0.0007 (7)	0.0014 (7)	0.0041 (7)
C4	0.0155 (9)	0.0173 (8)	0.0163 (9)	0.0002 (7)	0.0007 (7)	0.0030 (7)
C5	0.0213 (10)	0.0144 (8)	0.0204 (9)	0.0000 (7)	0.0028 (7)	0.0031 (7)
C6	0.0194 (9)	0.0159 (9)	0.0210 (9)	−0.0037 (7)	0.0022 (8)	−0.0002 (7)

### (I) µ-Oxalato-κ4O1,O2:O1',O2'-bis[[4-(2-aminoethyl)-1H-imidazole-κ2N3,N4](azido-κN1)copper(II)] Geometric parameters (Å, º)

**Table:**

Cu1—N4	1.9454 (16)	N6—C6	1.487 (2)
Cu1—N6	1.9904 (17)	N6—H6A	0.87 (3)
Cu1—O1	2.0088 (13)	N6—H6B	0.89 (3)
Cu1—O2	2.0148 (13)	C1—O1^i^	1.257 (2)
Cu1—N1	2.2679 (17)	C1—C1^i^	1.542 (4)
O1—C1^i^	1.257 (2)	C2—H2A	0.9500
O2—C1	1.254 (2)	C3—C4	1.363 (3)
N1—N2	1.185 (2)	C3—H3	0.9500
N2—N3	1.171 (2)	C4—C5	1.497 (3)
N4—C2	1.330 (2)	C5—C6	1.520 (3)
N4—C4	1.388 (2)	C5—H5A	0.9900
N5—C2	1.338 (2)	C5—H5B	0.9900
N5—C3	1.374 (3)	C6—H6C	0.9900
N5—H5	0.8800	C6—H6D	0.9900
			
N4—Cu1—N6	94.58 (7)	H6A—N6—H6B	102 (2)
N4—Cu1—O1	171.72 (6)	O2—C1—O1^i^	126.59 (17)
N6—Cu1—O1	88.11 (6)	O2—C1—C1^i^	116.9 (2)
N4—Cu1—O2	91.58 (6)	O1^i^—C1—C1^i^	116.48 (19)
N6—Cu1—O2	158.12 (7)	N4—C2—N5	110.11 (17)
O1—Cu1—O2	83.16 (5)	N4—C2—H2A	124.9
N4—Cu1—N1	98.24 (6)	N5—C2—H2A	124.9
N6—Cu1—N1	103.21 (7)	C4—C3—N5	106.62 (16)
O1—Cu1—N1	88.73 (6)	C4—C3—H3A	126.7
O2—Cu1—N1	96.63 (6)	N5—C3—H3A	126.7
C1^i^—O1—Cu1	111.73 (11)	C3—C4—N4	108.08 (16)
C1—O2—Cu1	111.40 (11)	C3—C4—C5	130.79 (17)
N2—N1—Cu1	111.39 (13)	N4—C4—C5	121.13 (16)
N3—N2—N1	178.6 (2)	C4—C5—C6	112.82 (16)
C2—N4—C4	106.89 (16)	C4—C5—H5A	109.0
C2—N4—Cu1	127.24 (13)	C6—C5—H5A	109.0
C4—N4—Cu1	125.82 (12)	C4—C5—H5B	109.0
C2—N5—C3	108.30 (16)	C6—C5—H5B	109.0
C2—N5—H5	125.8	H5A—C5—H5B	107.8
C3—N5—H5	125.8	N6—C6—C5	111.28 (16)
C6—N6—Cu1	120.15 (12)	N6—C6—H6C	109.4
C6—N6—H6A	108.7 (16)	C5—C6—H6C	109.4
Cu1—N6—H6A	106.6 (16)	N6—C6—H6D	109.4
C6—N6—H6B	108.9 (16)	C5—C6—H6D	109.4
Cu1—N6—H6B	108.7 (17)	H6C—C6—H6D	108.0

Symmetry code: (i) −*x*+1, −*y*+1, −*z*+1.

### (I) µ-Oxalato-κ4O1,O2:O1',O2'-bis[[4-(2-aminoethyl)-1H-imidazole-κ2N3,N4](azido-κN1)copper(II)] Hydrogen-bond geometry (Å, º)

**Table:**

*D*—H···*A*	*D*—H	H···*A*	*D*···*A*	*D*—H···*A*
N5—H5···N1^ii^	0.88	2.18	2.904 (2)	140
N5—H5···N3^iii^	0.88	2.69	3.258 (2)	124
N6—H6*A*···N3^iv^	0.87 (3)	2.29 (3)	3.099 (3)	154 (2)
N6—H6*B*···O1^v^	0.89 (3)	2.17 (3)	3.061 (2)	175 (2)
C3—H3···N3^vi^	0.95	2.54	3.476 (8)	169

Symmetry codes: (ii) −*x*+1, *y*+1/2, −*z*+3/2; (iii) *x*−1/2, *y*, −*z*+3/2; (iv) *x*−1/2, −*y*+1/2, −*z*+1; (v) −*x*+1, −*y*, −*z*+1; (vi) −*x*+1, *y*−1/2, −*z*+3/2.

### (II) µ-Oxalato-κ4O1,O2:O1',O2'-bis[[4-(2-aminoethyl)-1H-imidazole-κ2N3,N4](dicyanamido-κN1)copper(II)] Crystal data

**Table:**

[Cu_2_(C_2_O_4_)(C_2_N_3_)_2_(C_5_H_9_N_3_)_2_]	*F*(000) = 576
*M**_r_* = 569.50	*D*_x_ = 1.778 Mg m^−^^3^
Monoclinic, *P*2_1_/*c*	Mo *K*α radiation, λ = 0.71073 Å
*a* = 9.6816 (7) Å	Cell parameters from 87 reflections
*b* = 14.7236 (11) Å	θ = 2.0–28.0°
*c* = 7.4604 (6) Å	µ = 2.05 mm^−^^1^
β = 90.112 (1)°	*T* = 173 K
*V* = 1063.46 (14) Å^3^	Plate, blue
*Z* = 2	0.32 × 0.27 × 0.18 mm

### (II) µ-Oxalato-κ4O1,O2:O1',O2'-bis[[4-(2-aminoethyl)-1H-imidazole-κ2N3,N4](dicyanamido-κN1)copper(II)] Data collection

**Table:**

Bruker SMART CCD area-detector diffractometer	2374 independent reflections
Radiation source: fine-focus sealed tube	2172 reflections with *I* > 2σ(*I*)
Graphite monochromator	*R*_int_ = 0.061
ω scans	θ_max_ = 27.5°, θ_min_ = 2.1°
Absorption correction: analytical (SADABS; Bruker, 1998)	*h* = −12→10
*T*_min_ = 0.340, *T*_max_ = 0.503	*k* = −18→18
6331 measured reflections	*l* = −8→9

### (II) µ-Oxalato-κ4O1,O2:O1',O2'-bis[[4-(2-aminoethyl)-1H-imidazole-κ2N3,N4](dicyanamido-κN1)copper(II)] Refinement

**Table:**

Refinement on *F*^2^	Secondary atom site location: difference Fourier map
Least-squares matrix: full	Hydrogen site location: mixed
*R*[*F*^2^ > 2σ(*F*^2^)] = 0.034	H atoms treated by a mixture of independent and constrained refinement
*wR*(*F*^2^) = 0.096	*w* = 1/[σ^2^(*F*_o_^2^) + (0.0382*P*)^2^ + 1.0333*P*] where *P* = (*F*_o_^2^ + 2*F*_c_^2^)/3
*S* = 1.15	(Δ/σ)_max_ = 0.001
2374 reflections	Δρ_max_ = 0.34 e Å^−^^3^
198 parameters	Δρ_min_ = −0.42 e Å^−^^3^
55 restraints	Extinction correction: SHELXL2014 (Sheldrick, 2015), Fc^*^=kFc[1+0.001xFc^2^λ^3^/sin(2θ)]^-1/4^
Primary atom site location: structure-invariant direct methods	Extinction coefficient: 0.022 (2)

### (II) µ-Oxalato-κ4O1,O2:O1',O2'-bis[[4-(2-aminoethyl)-1H-imidazole-κ2N3,N4](dicyanamido-κN1)copper(II)] Special details

**Table:**

Geometry. All esds (except the esd in the dihedral angle between two l.s. planes) are estimated using the full covariance matrix. The cell esds are taken into account individually in the estimation of esds in distances, angles and torsion angles; correlations between esds in cell parameters are only used when they are defined by crystal symmetry. An approximate (isotropic) treatment of cell esds is used for estimating esds involving l.s. planes.
Refinement. All H atoms were positioned geometrically (C—H = 0.93/1.00 Å) and allowed to ride with *U*_iso_(H)= 1.2/1.5*U*_eq_(C). Methyl ones were allowed to rotate around the corresponding C—C.A C2H4 is disordered and was refined in three parts with their site occupation factors adding up to one. As a consequence of this disorder, the two protons on the adjacent N atom are disordered and also were calculated is three idealized positioned and were treated riding on their parent atom. SADI and RIGU were applied to the disorder geometry to maintain equivalent bond lengths of corresponding bonds.Proton H3 were obtained from a Difference Fourier map and refined freely.

### (II) µ-Oxalato-κ4O1,O2:O1',O2'-bis[[4-(2-aminoethyl)-1H-imidazole-κ2N3,N4](dicyanamido-κN1)copper(II)] Fractional atomic coordinates and isotropic or equivalent isotropic displacement parameters (Å^2^)

**Table:**

	*x*	*y*	*z*	*U*_iso_*/*U*_eq_	Occ. (<1)
Cu1	0.31334 (3)	0.56430 (2)	0.21722 (4)	0.02660 (15)	
O1	0.50672 (18)	0.51395 (14)	0.2322 (2)	0.0288 (4)	
O2	0.32980 (18)	0.53232 (14)	−0.0421 (2)	0.0294 (4)	
N1	0.3199 (2)	0.58973 (16)	0.4781 (3)	0.0274 (4)	
H1A	0.3702	0.5447	0.5303	0.033*	0.516 (3)
H1B	0.3682	0.6422	0.4933	0.033*	0.516 (3)
H1C	0.3102	0.5359	0.5368	0.033*	0.243 (3)
H1D	0.4057	0.6112	0.5044	0.033*	0.243 (3)
H1E	0.3862	0.5547	0.5302	0.033*	0.240 (3)
H1F	0.3426	0.6490	0.4961	0.033*	0.240 (3)
N2	0.1213 (2)	0.60545 (14)	0.1895 (3)	0.0235 (4)	
N3	−0.0839 (2)	0.62944 (17)	0.0764 (3)	0.0322 (5)	
H3	−0.145 (5)	0.634 (3)	−0.015 (7)	0.073 (14)*	
N4	0.4224 (3)	0.7083 (2)	0.1465 (4)	0.0516 (7)	
N5	0.6458 (3)	0.7761 (2)	0.0675 (4)	0.0409 (6)	
N6	0.7630 (3)	0.89650 (17)	0.2373 (3)	0.0360 (5)	
C1	0.5513 (2)	0.49455 (17)	0.0797 (3)	0.0236 (5)	
C2	0.1885 (7)	0.5987 (6)	0.5821 (12)	0.0267 (19)	0.516 (3)
H2A	0.2099	0.6155	0.7077	0.032*	0.516 (3)
H2B	0.1392	0.5399	0.5830	0.032*	0.516 (3)
C3	0.0970 (10)	0.6712 (6)	0.4982 (8)	0.0285 (19)	0.516 (3)
H3A	0.0213	0.6867	0.5818	0.034*	0.516 (3)
H3B	0.1520	0.7268	0.4765	0.034*	0.516 (3)
C2'	0.2169 (9)	0.6548 (8)	0.5547 (15)	0.031 (2)	0.243 (3)
H2'A	0.2272	0.7153	0.4985	0.037*	0.243 (3)
H2'B	0.2311	0.6613	0.6856	0.037*	0.243 (3)
C3'	0.0738 (14)	0.6165 (13)	0.5163 (10)	0.033 (4)	0.243 (3)
H3'A	0.0053	0.6442	0.5981	0.039*	0.243 (3)
H3'B	0.0736	0.5500	0.5358	0.039*	0.243 (3)
C2"	0.1832 (11)	0.5704 (13)	0.564 (3)	0.029 (4)	0.240 (3)
H2"A	0.1949	0.5669	0.6956	0.035*	0.240 (3)
H2"B	0.1480	0.5110	0.5214	0.035*	0.240 (3)
C3"	0.079 (3)	0.6445 (17)	0.5181 (13)	0.033 (6)	0.240 (3)
H3"A	0.1213	0.7048	0.5408	0.040*	0.240 (3)
H3"B	−0.0028	0.6384	0.5956	0.040*	0.240 (3)
C4	0.0362 (3)	0.6377 (2)	0.3221 (4)	0.0328 (6)	
C5	−0.0920 (3)	0.6523 (2)	0.2523 (4)	0.0332 (6)	
H5A	−0.1711	0.6739	0.3143	0.040*	
C6	0.0449 (3)	0.6013 (2)	0.0430 (4)	0.0345 (6)	
H6A	0.0770	0.5810	−0.0705	0.041*	
C7	0.5280 (3)	0.74143 (19)	0.1173 (4)	0.0320 (6)	
C8	0.7025 (3)	0.84044 (18)	0.1632 (3)	0.0283 (5)	

### (II) µ-Oxalato-κ4O1,O2:O1',O2'-bis[[4-(2-aminoethyl)-1H-imidazole-κ2N3,N4](dicyanamido-κN1)copper(II)] Atomic displacement parameters (Å^2^)

**Table:**

	*U*^11^	*U*^22^	*U*^33^	*U*^12^	*U*^13^	*U*^23^
Cu1	0.01728 (19)	0.0447 (2)	0.01783 (19)	0.00787 (12)	0.00023 (11)	−0.00297 (12)
O1	0.0178 (8)	0.0499 (11)	0.0187 (8)	0.0068 (7)	0.0004 (6)	−0.0011 (8)
O2	0.0172 (8)	0.0505 (11)	0.0204 (8)	0.0075 (7)	0.0008 (6)	−0.0015 (8)
N1	0.0223 (10)	0.0381 (12)	0.0218 (10)	0.0056 (9)	−0.0013 (8)	−0.0024 (9)
N2	0.0189 (9)	0.0293 (11)	0.0222 (10)	0.0040 (8)	0.0009 (7)	−0.0011 (8)
N3	0.0219 (10)	0.0457 (13)	0.0289 (11)	0.0071 (9)	−0.0016 (9)	0.0006 (10)
N4	0.0501 (16)	0.0543 (17)	0.0504 (16)	−0.0225 (14)	0.0002 (13)	0.0072 (14)
N5	0.0345 (12)	0.0526 (15)	0.0356 (13)	−0.0118 (11)	0.0026 (10)	−0.0124 (11)
N6	0.0357 (12)	0.0359 (13)	0.0365 (13)	−0.0036 (10)	−0.0028 (10)	0.0009 (11)
C1	0.0183 (10)	0.0333 (12)	0.0193 (11)	0.0009 (9)	−0.0004 (9)	0.0010 (9)
C2	0.029 (3)	0.030 (5)	0.021 (3)	0.005 (2)	0.007 (2)	−0.002 (3)
C3	0.031 (3)	0.028 (4)	0.027 (3)	0.010 (3)	0.001 (2)	−0.004 (2)
C2'	0.028 (4)	0.039 (6)	0.025 (5)	0.006 (4)	−0.001 (4)	−0.008 (4)
C3'	0.025 (5)	0.052 (10)	0.022 (5)	0.005 (5)	0.005 (4)	−0.013 (5)
C2"	0.031 (5)	0.044 (10)	0.012 (5)	0.011 (5)	0.000 (4)	−0.007 (6)
C3"	0.033 (8)	0.041 (14)	0.027 (6)	0.008 (7)	−0.004 (5)	−0.018 (5)
C4	0.0243 (12)	0.0475 (16)	0.0265 (12)	0.0116 (11)	0.0012 (10)	−0.0070 (11)
C5	0.0218 (12)	0.0462 (16)	0.0315 (13)	0.0110 (11)	0.0019 (10)	−0.0003 (12)
C6	0.0244 (12)	0.0556 (17)	0.0236 (12)	0.0098 (12)	−0.0021 (10)	−0.0032 (12)
C7	0.0346 (14)	0.0300 (13)	0.0315 (13)	0.0002 (11)	−0.0052 (11)	0.0028 (11)
C8	0.0248 (12)	0.0339 (13)	0.0261 (12)	0.0035 (10)	0.0008 (10)	0.0038 (10)

### (II) µ-Oxalato-κ4O1,O2:O1',O2'-bis[[4-(2-aminoethyl)-1H-imidazole-κ2N3,N4](dicyanamido-κN1)copper(II)] Geometric parameters (Å, º)

**Table:**

Cu1—N2	1.966 (2)	N6—C8	1.153 (4)
Cu1—N1	1.983 (2)	C1—O2^i^	1.249 (3)
Cu1—O2	1.9978 (18)	C1—C1^i^	1.557 (4)
Cu1—O1	2.0165 (17)	C2—C3	1.521 (7)
Cu1—N4	2.426 (3)	C2—H2A	0.9900
O1—C1	1.251 (3)	C2—H2B	0.9900
O2—C1^i^	1.249 (3)	C3—C4	1.521 (5)
N1—C2	1.497 (5)	C3—H3A	0.9900
N1—C2'	1.497 (6)	C3—H3B	0.9900
N1—C2"	1.498 (6)	C2'—C3'	1.522 (8)
N1—H1A	0.9100	C2'—H2'A	0.9900
N1—H1B	0.9100	C2'—H2'B	0.9900
N1—H1C	0.9100	C3'—C4	1.526 (6)
N1—H1D	0.9100	C3'—H3'A	0.9900
N1—H1E	0.9100	C3'—H3'B	0.9900
N1—H1F	0.9100	C2"—C3"	1.521 (8)
N2—C6	1.320 (3)	C2"—H2"A	0.9900
N2—C4	1.373 (3)	C2"—H2"B	0.9900
N3—C6	1.338 (3)	C3"—C4	1.524 (6)
N3—C5	1.357 (4)	C3"—H3"A	0.9900
N3—H3	0.91 (5)	C3"—H3"B	0.9900
N4—C7	1.154 (4)	C4—C5	1.362 (4)
N5—C7	1.304 (4)	C5—H5A	0.9500
N5—C8	1.306 (4)	C6—H6A	0.9500
			
N2—Cu1—N1	94.21 (9)	H2A—C2—H2B	108.2
N2—Cu1—O2	92.75 (8)	C4—C3—C2	110.7 (7)
N1—Cu1—O2	173.01 (8)	C4—C3—H3A	109.5
N2—Cu1—O1	175.44 (8)	C2—C3—H3A	109.5
N1—Cu1—O1	89.27 (8)	C4—C3—H3B	109.5
O2—Cu1—O1	83.74 (7)	C2—C3—H3B	109.5
N2—Cu1—N4	96.87 (10)	H3A—C3—H3B	108.1
N1—Cu1—N4	92.03 (10)	N1—C2'—C3'	107.3 (9)
O2—Cu1—N4	87.70 (10)	N1—C2'—H2'A	110.3
O1—Cu1—N4	85.92 (10)	C3'—C2'—H2'A	110.3
C1—O1—Cu1	110.86 (15)	N1—C2'—H2'B	110.3
C1^i^—O2—Cu1	111.54 (15)	C3'—C2'—H2'B	110.3
C2—N1—Cu1	120.0 (4)	H2'A—C2'—H2'B	108.5
C2'—N1—Cu1	118.4 (4)	C2'—C3'—C4	108.6 (8)
C2"—N1—Cu1	110.9 (8)	C2'—C3'—H3'A	110.0
C2—N1—H1A	107.3	C4—C3'—H3'A	110.0
Cu1—N1—H1A	107.3	C2'—C3'—H3'B	110.0
C2—N1—H1B	107.3	C4—C3'—H3'B	110.0
Cu1—N1—H1B	107.3	H3'A—C3'—H3'B	108.4
H1A—N1—H1B	106.9	N1—C2"—C3"	110.5 (17)
C2'—N1—H1C	107.7	N1—C2"—H2"A	109.5
Cu1—N1—H1C	107.7	C3"—C2"—H2"A	109.5
C2'—N1—H1D	107.7	N1—C2"—H2"B	109.5
Cu1—N1—H1D	107.7	C3"—C2"—H2"B	109.5
H1C—N1—H1D	107.1	H2"A—C2"—H2"B	108.1
C2"—N1—H1E	109.5	C2"—C3"—C4	110.4 (15)
Cu1—N1—H1E	109.5	C2"—C3"—H3"A	109.6
C2"—N1—H1F	109.5	C4—C3"—H3"A	109.6
Cu1—N1—H1F	109.5	C2"—C3"—H3"B	109.6
H1E—N1—H1F	108.0	C4—C3"—H3"B	109.6
C6—N2—C4	106.1 (2)	H3"A—C3"—H3"B	108.1
C6—N2—Cu1	126.94 (18)	C5—C4—N2	109.1 (2)
C4—N2—Cu1	126.83 (17)	C5—C4—C3	129.0 (4)
C6—N3—C5	108.2 (2)	N2—C4—C3	120.2 (4)
C6—N3—H3	120 (3)	C5—C4—C3"	127.2 (10)
C5—N3—H3	132 (3)	N2—C4—C3"	123.3 (10)
C7—N4—Cu1	142.8 (3)	C5—C4—C3'	127.6 (6)
C7—N5—C8	119.7 (3)	N2—C4—C3'	118.1 (7)
O2^i^—C1—O1	126.6 (2)	N3—C5—C4	106.0 (2)
O2^i^—C1—C1^i^	116.7 (3)	N3—C5—H5A	127.0
O1—C1—C1^i^	116.7 (3)	C4—C5—H5A	127.0
N1—C2—C3	110.1 (7)	N2—C6—N3	110.6 (2)
N1—C2—H2A	109.6	N2—C6—H6A	124.7
C3—C2—H2A	109.6	N3—C6—H6A	124.7
N1—C2—H2B	109.6	N4—C7—N5	174.2 (3)
C3—C2—H2B	109.6	N6—C8—N5	173.6 (3)

Symmetry code: (i) −*x*+1, −*y*+1, −*z*.

### (II) µ-Oxalato-κ4O1,O2:O1',O2'-bis[[4-(2-aminoethyl)-1H-imidazole-κ2N3,N4](dicyanamido-κN1)copper(II)] Hydrogen-bond geometry (Å, º)

**Table:**

*D*—H···*A*	*D*—H	H···*A*	*D*···*A*	*D*—H···*A*
N1—H1*E*···O1^ii^	0.91	2.29	3.131 (3)	154
N1—H1*F*···N4^iii^	0.91	2.50	3.377 (4)	161
N3—H3···N6^iv^	0.91 (5)	2.10 (5)	2.954 (3)	157 (4)
C2—H2*A*···O2^v^	0.99	2.52	3.277 (1)	133

Symmetry codes: (ii) −*x*+1, −*y*+1, −*z*+1; (iii) *x*, −*y*+3/2, *z*+1/2; (iv) *x*−1, −*y*+3/2, *z*−1/2; (v) *x*, *y*, *z*+1.
